# The History of the Middlesex Hospital during the First Century of Its Existence

**Published:** 1846-01

**Authors:** 


ART. XI.—*The History of the Middlesex Hospital during the first Century of it existence.* By ERASMUS WILSON, F.R.S.

This is a work most creditable to the author. It is almost unique of its kind; at least we have never seen any account of a British hospital at all to be compared with it in fulness and accuracy of detail. If in addition to the economical history, Mr. Wilson could have given us the medical history of the hospital—that is, a complete report of the diseases admitted, with their events, &c., during the same period, he would have left us nothing to desire. We entirely assent to the author's estimate of the probable influence of the present work on those into whose hands it may fall.

"Certainly, if a picture of the admirable management of a truly benovolent institution be calculated to make an impression upon the human mind,—if the testimony of an upright and conscientious spirit of action have any weight with the charitable and the humane, the conduct of the Governors of the Middlesex Hospital, during a century of usefulness, cannot be otherwise than favorably estimated." (p. x.)

